# Higher levels of thyroxine may predict a favorable response to donepezil treatment in patients with Alzheimer disease: a prospective, case–control study

**DOI:** 10.1186/s12868-018-0436-x

**Published:** 2018-06-22

**Authors:** Yu San Chang, Yu Hsuan Wu, Chin Jen Wang, Shu Hui Tang, Hsiang Lan Chen

**Affiliations:** 1 Kaohsiung Municipal Kai-Syuan Psychiatric Hospital, No. 130, Kai-Syuan, 2nd Road, Ling-Ya District, Kaohsiung, 802 Taiwan; 2 Faculty of Nursing Department, Meiho University, No. 23, Pingguang Road, Neipu, Pingtung Taiwan

**Keywords:** Alzheimer disease, Cortisol, Thyroid hormone, Donepezil, Cholinesterase inhibitors

## Abstract

**Background:**

Cholinergic hypothesis has been advanced as an etiology of Alzheimer disease (AD) on the basis of the presynaptic deficit found in the diseased brains, and cholinesterase inhibitors (ChEIs) are the treatment of choice for these patients. However, only about half of treatment efficacy was found. Because increasing evidence supports an extensive interrelationship between thyroid hormones (THs), cortisol level and the cholinergic system, the aim of the present study was to evaluate thyroid function and cortisol level in patients with mild to moderate AD before and after ChEIs treatment, and to identify possible variations in response. This was a prospective, case–control, follow-up study. Levels of cortisol and THs were evaluated in 21 outpatients with mild to moderate AD and 20 normal controls. All patients were treated with 5 mg/day of donepezil (DPZ) and were reevaluated after 24–26 weeks of treatment.

**Results:**

The patients had worse cognitive function, higher cortisol level, and lower levels of triiodothyronine (T_3_) and its free fraction than the controls. There were no significant differences in global cognitive function or cortisol level after treatment, however, significant reductions in T_3_ and thyroxin (T_4_) levels were observed. Responders had higher levels of T_4_ than non-responders, followed by a significant reduction after treatment.

**Conclusions:**

These results suggest that relatively higher levels of T_4_ may predict a favorable response to DPZ treatment. Further studies are warranted to confirm the relationship between THs and ChEIs therapy in AD and to explore new therapeutic strategies. On the other hand, cortisol levels are more likely to respond to interventions for stress-related neuropsychiatric symptoms in patients with AD rather than ChEIs treatment. Further studies are warranted to investigate the association between cortisol level and the severity of stress-related neuropsychiatric symptoms in patients with AD.

## Background

Alzheimer disease (AD) is a degenerative disorder of the central nervous system (CNS), for which the cholinergic hypothesis has been proposed to be an etiology on the basis of presynaptic deficits found in diseased brains. Increasing evidence also supports an extensive interrelationship between thyroid hormones (THs), cortisol level and the cholinergic system. Thyroxin (T_4_) has been shown to modulate choline acetyltransferase activity, and triiodothyronine (T_3_) has been shown to negatively regulate the expression of amyloid precursor protein in the brain [1, 2]. Regarding cortisol level, Degroot et al. [3] reported a parallel increase in levels of plasma cortisol and hippocampal acetylcholine (Ach) after acute stress. In addition, corticosterone administration, which mimics the increase in the plasma corticosterone concentration produced by stress, has also been shown to induce hippocampal Ach [4, 5].

Currently, the most common drug treatments are based on the cholinergic hypothesis. This hypothesis states that a lower production of acetylcholine, a neurotransmitter, leads to AD, and thus cholinesterase inhibitors (ChEIs) are the recommended therapeutic option [6]. However, only about half of treatment efficacy was found [7], and the reasons for nonresponse remains largely unknown. The study aimed to evaluate thyroid function and cortisol level in patients with mild to moderate AD before and after ChEIs treatment, and to identify possible variations in response.

## Methods

### Participants

This was a prospective, case–control, 24- to 26-week follow-up study. Twenty one outpatients (7 men and 14 women; mean age: 78.5 ± 5.7 years; mean years of education: 5.0 ± 5.1 years) with mild to moderate AD according to the Clinical Dementia Rating scale [8] and a Mini–Mental State Examination (MMSE) [9] score of 10–24 [10] were enrolled. The diagnostic criteria were based on the Diagnostic and Statistical Manual of Mental Disorders, 4th ed. (DSM-IV) [11], the National Institute of Neurological and Communicative Disorders and Stroke, and the Alzheimer’s Disease and Related Disorders Association [12] for probable AD. All of the patients underwent a neurological examination, neuroimaging and laboratory workup to rule out other treatable causes of dementia. Twenty normal controls (9 males and 11 females; mean age: 77.5 ± 4.4 years; mean years of education: 6.6 ± 4.4 years) were also recruited from the hospital and general community.

None of the participants had a history of stroke, epilepsy, thyroid disease, pathological levels of urea or creatinine, other significant psychiatric diseases, either using or had used glucosteroid supplements, or any CNS-active drug treatment. The study protocol had the approval of the Ethics Committee of Kaohsiung Municipal Kai-Syuan Psychiatric Hospital. Written informed consent was obtained from all participants and authorized caregivers after the study had been fully explained and the potential risks and benefits had been discussed.

### Procedures

Neuropsychological assessments, thyroid function tests and serum levels of cortisol were obtained at a screening appointment for all participants. Researches [6] showed patients with AD had most benefit from ChEIs treatment for cognition within the first 6 months followed by a gradual decline thereafter [6]. Therefore, we chose 24–26 weeks as the study period. All patients were treated with 5 mg/day of donepezil (DPZ) during the study period and underwent neuropsychological assessments at 24–26 weeks. At that time, thyroid status and serum levels of cortisol were also measured. The neuropsychological assessment assessor was blinded to the results of THs and cortisol levels.

According to the Bureau of National Health Insurance in Taiwan, it is necessary to evaluate cognitive function at least every 1 year after the initial administration of DPZ to apply for continuing prescriptions, and patients with MMSE scores of 2 or more points lower than the pretreatment level will not be eligible for reimbursements for continuing prescriptions. Therefore, response was a priori defined as a stabilization or improvement in MMSE score in this study. The patients with MMSE scores of 2 or more points lower than the pretreatment level were defined as being non-responders.

### Laboratory assessments

Fasting blood samples (5 ml) were collected by venipuncture between 8:00 and 9:00 a.m. to determine levels of thyroid stimulating hormone (TSH), T_3_, T_4_, free fractions (FT_3_ and FT_4_), and cortisol. Serum was separated immediately after blood collection, and the samples were stored at − 20 °C until analysis. Quantitative determination of cortisol and thyroid status in the serum was measured using paramagnetic particles in a chemiluminescent immunometric assay using a Beckman Access system (Beckman Coulter Inc., Fullerton, CA, USA) for cortisol level and an Abbott I2000 system (Abbott Ireland Diagnostics Division, Longford, Ireland) for thyroid status. The lower limits of detection were 0.0025 mIU/l for TSH, 25.0 ng/dl for T_3_, < 1.0 μg/dl for T_4_, < 1.0 pg/ml for FT_3_, 0.4 ng/dl for FT_4_, and 0.4 μg/dl for cortisol. The intra-assay coefficient of variation averaged 5% for each item.

### Statistical analysis

Sex distribution between groups was compared using the χ^2^ test. Group differences in age, years of education, neuropsychological, thyroid status and cortisol level assessments were evaluated using the Student’s *t* test. Repeated measures ANOVA were used to compare the follow-up measurements with baseline data in terms of neuropsychological assessments, thyroid status and cortisol level, and age and years of education were used as covariates for neuropsychological assessments. To identify differences that may have resulted in variations in response, differences in baseline of those significant follow-up hormone measurements were compared between the responders and non-responders using the Student’s *t* test. In responders, the significant hormone items were also compared the follow-up measurements with baseline data using repeat measures ANOVA. A *p* value less than 0.05 was considered to be statistically significant. The partial eta-squared value was calculated for the follow-up measurements for each variable.

## Results

**Fig. 1:**
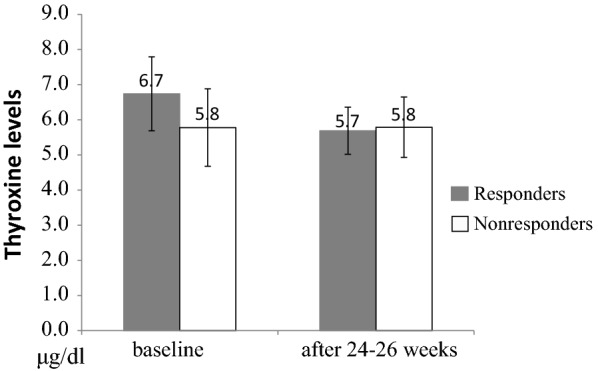
Thyroxine levels (μg/dl) in responders (gray bars) and nonresponders (white bars) to donepezil therapy at baseline and 24–26 weeks after treatment. The responders (n = 13) had with a higher baseline level of T_4_ compared with the non-responders (n = 8) (t(19) = 2.29, *p* = .033) and a significant reduction in T_4_ level after 24–26 weeks of donepezil treatment (F(1,12) = 13.13, *p* = .003)

There were no significant differences in sex, age, and years of education between the patients with AD and the controls. The baseline neuropsychological and hormone assessments for both groups are summarized in Table 1. Compared to the controls, the patients with AD had a higher cortisol level, lower levels of T_3_ and FT_3_, and a lower MMSE scores. There were no significant differences in MMSE scores and cortisol level before and after 24–26 weeks of treatment with DPZ. However, significant reductions in T_3_ and T_4_ levels were observed (Table 2). There was no significant difference in baseline level of T_3_ between the responders (61.9%) and non-responders (t(19) = − 1.65, *p* = .115), and no significant reduction was found in T_3_ level after 24–26 weeks of DPZ treatment in the responders (F(1, 12) = 3.01, *p* = .109). However, the responders had with a higher baseline level of T_4_ compared with the non-responders (t(19) = 2.29, *p* = .033) and a significant reduction in T_4_ level after 24–26 weeks of DPZ treatment (F(1,12) = 13.13, *p* = .003) (Fig. 1). The reduction ratio was about 85.3 ± 16.0% of baseline T_4_ levels.

**Table 1:** Demographic and clinical characteristics between in patients with Alzheimer disease (AD) and controls

Variables (mean ± SD)	Controls	AD patients	*t*(39) or χ^2^ (1) values	*p* value
n (M/F)	20 (9/11)	21 (7/14)	0.59	.530
Age (year)	77.5 ± 4.4	78.5 ± 5.7	− 0.67	.504
Years of education	6.6 ± 4.4	5.0 ± 5.1	1.07	.293
MMSE scores	27.5 ± 1.9	16.1 ± 5.4	8.93	< .001
Cortisol (μg/dl)	7.8 ± 3.7	10.2 ± 2.9	− 2.34	.025
Thyrotropin (mIU/l)	1.4 ± 0.7	1.4 ± 0.9	− 0.01	.996
Total T_3_ (ng/dl)	94.8 ± 10.2	85.5 ± 8.6	3.18	.003
Free T_3_ (pg/ml)	2.5 ± 0.4	2.3 ± 0.3	2.56	.015
Total T_4_ (μg/dl)	6.3 ± 1.1	6.4 ± 1.0	− 0.33	.747
Free T_4_ (ng/dl)	1.0 ± 0.1	1.0 ± 0.1	− 0.23	.818

*MMSE* Mini–Mental State Examination

**Table 2:** Neuropsychological function and hormone levels in patients at baseline and after administered donepezil for 24–26 weeks (n = 21)

Variables (mean ± SD)	Baseline	Follow up	F(1,18)^a^/F(1, 20)	*p* value	η_p_^2^ (%)
MMSE scores^a^	16.1 ± 5.4	17.1 ± 5.2	0.25	.624	1.4
Cortisol (μg/dl)	10.2 ± 2.9	10.6 ± 1.8	0.45	.511	2.2
Thyrotropin (mIU/l)	1.4 ± 0.9	1.4 ± 1.0	0.42	.523	2.1
Total T_3_ (ng/dl)	85.5 ± 8.6	81.4 ± 10.1	7.57	.012	27.4
Free T_3_ (pg/ml)	2.3 ± 0.3	2.2 ± 0.3	1.52	.233	7
Total T_4_ (μg/dl)	6.4 ± 1.0	5.7 ± 1.0	7.12	.015	26.3
Free T_4_ (ng/dl)	1.0 ± 0.1	1.0 ± 0.1	0.07	.800	0.3

*MMSE* Mini–Mental State Examination

^a^Age and years of education as covariates

## Discussion

In this study, the patients with AD had a statistically significantly higher cortisol level, lower levels of T_3_ and FT_3_, and a decline in global cognition compared with the controls. There were no significant differences in global cognitive function as measured by the MMSE and cortisol level after treatment, however significant reductions in T_3_ and T_4_ levels were observed. In addition, the responders had a higher level of T_4_ than the non-responders, followed by a significant reduction after treatment.

Laboratory studies have implicated a relationship between THs and factors associated with the pathogenesis of AD, including β-amyloid (Aβ) deposition and neuronal apoptosis. Circulation T_4_ are the major form of the THs which be transported into the brain by transthyretin [13]. T_4_ is then converted to the active formT_3_ and inactive rT_3_ by deiodinase. T_3_ has been shown to negatively regulate the expression of the amyloid precursor protein gene [2], T_4_ has been shown to modulate choline acetyltransferase activity [1], and transthyretin has been shown to create soluble Aβ complexes [14]. Compared with age-matched controls [15], the level of transthyretin has been reported to be lower in the cerebrospinal fluid (CSF) of patients with AD, suggesting a possible reduction in T_4_ transport into the brain in patients with AD. However, most T_3_ within the brain is produced locally by the intracerebral conversion of T_4_ to T_3_ [16]. In addition, increased rT_3_ levels and an increased rT_3_ to T4 ratio were also found in the CSF of AD patients [16]. Taken together, these findings suggest abnormal intracerebral THs metabolism and possibly brain hypothyroidism in AD [17]. Even though circulating THs do not properly reflect the bioactive portion of CNS activity, the lower levels of T_3_ and FT_3_ in our results also suggest a link between abnormalities in the hypothalamic–pituitary–thyroid axis and dementia.

High levels of glucocorticoid receptors have been reported in the hippocampus where is supposed to engage in the negative-feedback of glucocorticoid secretion [18]. However degeneration of the hippocampus is a prominent characteristic of AD [19], the loss of hippocampal cells can lead to hypercortisolemia, and this can increase degeneration of the hippocampus as the disease progresses. We also found higher levels of cortisol in the patients with AD than in the controls.

In the current, significant reductions in T_3_ and T_4_ levels were observed 24–26 weeks after DPZ treatment, indicating a relationship between THs and treatment. Thyroid glands are known to secrete about 80–90% of T_4_ and about 10–20% of T_3_, and that this process is regulated by TSH which is released by the anterior pituitary gland. Deiodinase enzymes in peripheral tissues convert T_4_ to T_3_ and rT_3_, and this is a major source of both rT_3_ (95%) and T_3_ (87%) in peripheral tissues [20]. However, thyroid activity is influenced by both neuroendocrine control of TSH release and also various neurotransmitters including Ach. Maayan et al. [21] reported that Ach possible involved of muscarinic receptors in the thyroid to inhibit TSH- induced T_4_ release. In addition, numerous acetylcholinesterase-positive nerve fibers, probably cholinergic, were found in the thyroid [22]. Therefore, our results of decreased T_3_ and T_4_ levels after treatment implicated the effect of Ach on the thyroid gland. However, there were no significant changes in TSH level after treatment in our study.

ChEIs treatment in AD has been reported to be most beneficial with regards to cognition after 3–4 months of administration [23], followed by a gradual decline after 24–36 weeks of treatment [6, 24]. DPZ is a ChEIs that has also been shown to exhibit a significantly greater improvement in cognitive function within 3 months of 5 mg/day administration, and to maintain a response defined by stabilization of cognitive function for 1 year [25]. Accordingly, the global cognitive function was improved after treatment in this study but did not have statistically significant. This may be because of the small sample size, or because we did not perform evaluations during the period of maximum benefit.

Many studiers have reported interactions between glucocorticoids and Ach in the brain [26–28]. Stress-induced responses not only activate the hypothalamic–pituitary–adrenal (HPA) axis but also the septo-hippocampal cholinergic pathway. Activation of the HPA axis leads to the release of corticosterone, and activation of the septo-hippocampal cholinergic pathway results in an increase in Ach in the hippocampus. Ach also mediates neuroendocrine, emotional, and physiological responses by stimulating the HPA axis. Thus, the hippocampus in tandem with basal forebrain cholinergic pathways is involved in regulating HPA axis stress responses. Hence, neurodegeneration of cholinergic neurons in patients with AD makes them vulnerable to stress, resulting in cognitive impairment.

In this study, we found no statistical difference in cortisol levels after DPZ treatment. A previous large prospective study did not find a relationship between cortisol levels and the risk of developing AD, and concluded that dysregulation of the HPA axis in patients with AD seemed to be a consequence rather than a cause of AD [29, 30]. In addition, other studies have reported that chronic exposure to high levels of endocrine glucocorticoids probably contributes to the intensification of neuropsychiatric symptoms, particularly in stress-related symptoms such as depression and anxiety [31–33]. It is therefore possible that cortisol levels in patients with AD would be more likely to respond to interventions for stress-related neuropsychiatric symptoms rather than ChEIs treatment. This may be why we did not find a relationship between cortisol levels and the effect of DPZ treatment in patients with AD.

In our results, the responders had a higher level of T_4_ than the non-responders, followed by a significant reduction after treatment. Although THs in the CNS depend almost entirely on the uptake of T_4_ and its intracellular deiodination to the active compound T_3_ [16], deiodinase enzymes have also been shown to convert T_4_ to T_3_ in peripheral tissues [20]. Thus, the reduction of serum T_4_ levels, as seen during DPZ treatment, may not only be due to enhanced conversion of T_4_ to T_3_ in the CNS. However, many neurotropic medications such as antidepressants, anticonvulsants, neuroleptics and benzodiazepines may affect deiodinase activity and THs concentration in the CNS [34]. Therefore, the effect of DPZ beyond that of ChEIs may involve the upregulation of deiodinase activity in the brain. Alternatively, the responders to DPZ presented with higher baseline T_4_ levels before treatment and showed a significant reduction after treatment, which may indicate that a higher level of T_4_ characterizes a subgroup of AD patients who would be expected to benefit from ChEIs treatment.

There are several limitations to the present study. First, the small sample size limits the interpretation of the findings. Second, circulating hormone assessments do not properly reflect the bioactive portion involved in the CNS. Third, we did not record body mass index or concomitant diseases such as diabetes mellitus and hypertension, all of which could have affected the levels of THs and cortisol. Fourth, the study lacks supporting epidemiological data to make an association between THs and the expected favorable therapeutic response to DPZ treatment. However, the clinical relevance of the preliminary findings provide information to identify possible variations in response. Finally, the study was restricted to DPZ, and thus the findings cannot be extrapolated to other ChEIs.

## Conclusions

In summary, we found that the patients with AD had no significant differences in cortisol levels after 24–26 weeks of treatment with DPZ, but significant reductions in T_3_ and T_4_ levels. The responders had a higher level of T_4_ than the non-responders, followed by a significant reduction after DPZ treatment. These results indicate that relatively higher levels of T_4_ may predict a favorable response to DPZ treatment. Further studies are warranted to confirm the relationship between THs and ChEIs therapy in AD and to explore new therapeutic strategies. On the other hand, cortisol levels are more likely to respond to interventions for stress-related neuropsychiatric symptoms in patients with AD rather than ChEIs treatment. Further studies are warranted to investigate the association between cortisol level and the severity of stress-related neuropsychiatric symptoms in patients with AD.

## Abbreviations

Ach: acetylcholine
AD: Alzheimer disease
Aβ: β-amyloid
ChEIs: cholinesterase inhibitors
CNS: central nervous system
CSF: cerebrospinal fluid
DPZ: donepezil
FT_3_: free fractions of triiodothyronine
FT_4_: free fractions of thyroxin
T_3_: triiodothyronine
T_4_: thyroxin
THs: thyroid hormones
TSH: thyroid stimulating hormone
HPA: hypothalamic–pituitary–adrenal
MMSE: Mini–Mental State Examination

## References

[1] Hayashi M, Patel AJ (1987). An interaction between thyroid hormone and nerve growth factor in the regulation of choline acetyltranserase activity in neuronal cultures derived from the septal-diagonal band region of the embryonic rat brain. Brain Res.

[2] Belandia B, Latasa MJ, Villa A, Pascual A (1998). Thyroid hormone negatively regulates the transcriptional activity of the β-amyloid precursor protein gene. J Biol Chem.

[3] Degroot A, Wade M, Salhoff C, Davis RJ, Tzavara ET, Nomikos G (2004). Exposure to an elevated platform increases plasma corticosterone and hippocampal acetylcholine in the rat: reversalbychlordiazepoxide. Eur J Pharmacol.

[4] Gilad GM, Mahon BD, Finkelstein Y, Koffler B, Gilad VH (1985). Stress-induced activation of the hippocampal cholinergic system and the pituitary- adrenocorticalaxis. Brain Res.

[5] Imperato A, Puglisi-Allegra S, Casolini P, Zocchi A, Angelucci L (1989). Stress- induced enhancement of dopamine and acetylcholine release in limbic structures: role of corticosterone. Eur J Pharmacol.

[6] Emilien G, Beyreuther K, Masters CL, Maloteaux JM (2000). Prospects for pharmacological intervention in Alzheimer disease. Arch Neurol.

[7] Moghul S, Wilkinson D (2001). Use of acetylcholinesterase inhibitors in Alzheimer’s disease. Exp Rev Neurother.

[8] Hughes CP, Berg L, Danziger WL, Coben LA, Martin RL (1982). A new clinical scale for the staging of dementia. Br J Psychiatry.

[9] Liu HC, Lin KN, Teng EL, Wang SJ, Fuh JL, Guo NW (1995). Prevalence and subtypes of dementia in Taiwan: a community survey of 5297 individuals. J Am Geriat Soc.

[10] Wang CY, Hua MS, Chiu MJ, Chu YC, Chen ST, Yip PK (2003). The comparison between Mini–Mental State Examination (MMSE) and Clinical Dementia Rating scale (CDR) in evaluating patients with Alzheimer’s disease. Taiwan J Psychiatry.

[11] American Psychiatric Association (1994). Diagnostic and statistical manual of mental disorders (DSM-IV).

[12] McKhann G, Drachman D, Folstein M, Katzman R, Price D, Stadlan EM (1984). Clinical diagnosis of Alzheimer’s disease: report of the NINCDS-ADRDA Work Group under the auspices of Department of Health and Human Services Task Force on Alzheimer’s disease. Neurology.

[13] Robbins J, Lakshmanan M (1992). The movement of thyroid hormones in the central nervous system. Acta Med Austriaca.

[14] Regelson W, Harkins SW (1997). Amyloid is not a tombstone. Ann NY Acad Sci.

[15] Serot JM, Christmann D, Dubost T, Couturier M (1997). Cerebrospinal fluid transthyretin: aging and late onset Alzheimer’s disease. J Neurol Neurosurg Psychiatry.

[16] Costa A, Arisio R, Benedetto C, Bertino E, Fabris C, Giraudi G (1991). Thyroid hormones in tissues from human embryos and fetuses. J Endocrinol Invest.

[17] Sampaolo S, Campos-Barros A, Mazziotti G, Carlomangno S, Sannino V, Amato G (2005). Increased cerebrospinal fluid levels of 3,3′,5′- triiodothyronine in patients with Alzheimer’s disease. J Clin Endocrinol Metab.

[18] Umegaki H, Ikari H, Nakahata H, Endo H, Suzuki Y, Ogawa O (2000). Plasma cortisol levels in elderly female subjects with Alzheimer’s disease: a cross-sectional and longitudinal study. Brain Res.

[19] Markesbery WR (1997). Neuripathological criteria for the diagnosis of Alzheimer’s disease. Neurobiol Aging.

[20] Nussey S, Whitehead N (2001). The thyroid gland in endocrinology: an integrated approach.

[21] Maayan ML, Volpert EM, From A (1983). Acetylcholine and norepinephrine: compared actions thyroid metabolism. Endocrinology.

[22] Grunditz T, Sundler F, Braverman LE, Utiger RD (1996). Autonomic nervous control: adrenergic, cholinergic, and peptidergic regulation. Werner and Ingbar’s the thyroid.

[23] Kapaki E, Paraskevas GP (2005). The cognitive effects of cholinesterase inhibitor treatment in every-day practice. Curr Med Res Opin.

[24] Rocca P, Cocuzza E, Marchiaro L, Bogetto F (2002). Donepezil in the treatment of Alzheimer’s disease long-term efficacy and safety. Prog Neuropsychopharmacol Biol Psychiatry.

[25] Evans M, Ellis A, Watson D, Chowdhury T (2000). Sustained cognitive improvement following treatment of Alzheimer’s disease with donepezil. Int J Geriatr Psychiatry.

[26] Newman MB, Nazian SJ, Sanberg PR, Diamond DM, Shytle RD (2001). Corticosterone- attenuating and anxiolytic properties of mecamylamine in the rat. Prog Neuropsychopharmacol Biol Psychiatry.

[27] Mora F, Segovia G, DelArco A, deBlas M, Garrido P (2012). Stress, neurotransmitters, corticosterone and body-brain integration. Brain Res.

[28] Paul S, Jeon WK, Bizon JL, Han JS (2015). Interaction of basal forebrain cholinergic neurons with the glucocorticoid system in stress regulation and cognitive impairment. Front Aging Neurosci.

[29] Schrijvers EM, Direk N, Koudstaal PJ, Kirschbaum C, Hofman A (2011). Associations of serum cortisol with cognitive function and dementia: the Rotterdam Study. J Alzheimers Dis.

[30] Gil-Bea FJ, Aisa B, Solomon A, Solas M, del Carmen Mugueta M, Winblad B (2010). HPA axis dysregulation associated to apolipoprotein E4 genotype in Alzheimer’s disease. J Alzheimers Dis.

[31] Landfield PW, Blalock EM, Chen KC, Porter NM (2007). A new glucocorticoid hypothesis of brain aging: implications for Alzheimer’s disease. Curr Alzheimer Res.

[32] Ricci S, Fuso A, Ippoliti F, Businaro R (2012). Stress-induced cytokines and neuronal dysfunction in Alzheimer’s disease. J Alzheimers Dis.

[33] Mah L, Szabuniewicz C, Fiocco AJ (2016). Can anxiety damage the brain?. Curr Opin Psychiatry.

[34] Baumgartner A (2000). Thyroxine and the treatment of affective disorders: an overview of the results of basic and clinical research. Int J Neuropsychopharmacol.

